# Supplementary material for: The operational impact of deploying SARS-CoV-2 vaccines in countries of the WHO African Region
Source: medRxiv. 2020 Nov 1:2020.08.13.20147595. Originally published 2020 Aug 14. Preprint. [Version 2] doi: 10.1101/2020.08.13.20147595 (PMC7430629; doi:10.1101/2020.08.13.20147595)
Supplement: Supplement 2020 [file 93088-2020.08.13.20147595-1.pdf]

Online Supplement

The operational impact of deploying SARS-CoV-2 vaccines in countries of the WHO African Region

Supplemental Table 1. Routine and SARS-CoV-2 vaccines, schedules, and cold storage volume per dose

	**Schedule[1]**	**Cold storage tertiary packaging volume per dose (mL)[2,3]**	**Cold storage secondary packaging volume per dose (mL)[2,3]**
Routine infant immunization			
Bacille Calmette-Guerin	Birth dose	3.6	1.3
Diphtheria-tetanus-pertussis-hepatitis B-Haemophilus influenzae type b (pentavalent)	6, 10 and 14 weeks	51.3	4.0
Polio (Inactivated)	6, 10 and 14 weeks	71.4	4.4
Pneumococcal (Conjugate)	6, 10 and 14 weeks	40.1	3.5
Rotavirus	6 and 10 weeks	34.7	7.4
Measles-rubella	9-12 months, 13-24 months	26.2	3.0
Tetanus-diphtheria	13-24 months	11.0	2.3
Meningococcal A (Conjugate)	13-24 months	14.6	2.1
Yellow fever	13-24 months	5.0	2.9
Routine children immunization			
HPV[4] (girls only)	2 doses from 9-14 years	7.6	5.7
Tetanus-diphtheria	9-14 years	11.0	2.3
SARS-CoV-2 immunization targeting risk groups			
≥65 years	2 doses, 1 month apart	12.9	5.2
Healthcare workers	2 doses, 1 month apart	12.9	5.2
Chronic diseases	2 doses, 1 month apart	12.9	5.2

Notes:

1. Vaccines and schedules are from WHO Immunization Tables except SARS-CoV-2 vaccines. [1]
2. Tertiary and secondary packaging volumes per dose from WHO Prequalified Vaccines Database with the exception of SARS-CoV-2 vaccines.[2] All data presented for multidose formulations.
3. SARS-CoV-2 cold storage volume per dose use prequalified multidose vial influenza vaccines as a proxy.
4. HPV vaccination programs generally target girls only aged 9 through 14 years.[1] For this simulation, we assumed the full HPV immunization series was given to girls 9 years of age.

Supplemental Table 2. Study assumptions

	Routine immunization assumption	SARS-CoV-2 immunization assumption	Comments
**Vaccines**	WHO prequalified vaccines in multidose vial presentation given per WHO recommended schedule.[14]	SARS-CoV-2 vaccine in multidose vial presentation given in a two vaccination series, one month apart.	Given that there are no licensed SARS-CoV-2 vaccines, dose assumptions rely on dose schedule of SARS-CoV-2 vaccine clinical trials in the United States to date, and the volume analyses use total tertiary packaging volume per dose of WHO prequalified influenza vaccines as a proxy.
**Packaging**	Tertiary packaging at the national level<br>Secondary packaging at all subnational levels	Tertiary packaging at national level<br>Secondary packaging at all subnational levels	National level vaccine volume analyses use total tertiary packaging volume required per dose (the unit for international transport), defined as the volume of the container holding cartons which contain vaccine vials divided by the total doses contained.<br>Subnational levels vaccine volume analysis use total secondary packaging volume required per dose, defined as the volume of cartons which contain vaccine vials divided by the total doses contained.
**Storage Temperature**	2° to 8°C	2° to 8°C	
**Coverage**	90% of target group	90% of target group	
**Target groups**	WHO recommended ages	Chronic diseases (any age)<br>Persons ≥ 65 years<br>Healthcare workers	Adults with chronic disease and older adults are at increased risk for severe SARS-CoV-2 disease.[3] Health care workers are at increased risk for SARS-CoV-2 infection and disease.[3]
**Strategy**	Year round	Four month mass vaccination campaign	Once pandemic vaccines are available, there will be an imperative to deliver them expeditiously.
**Wastage multidose vials**	25%	7.5%	Vaccine wastage is the doses that are lost or unused. Routine immunization inputs are from WHO guidance while SARS-CoV-2 inputs are from WHO Vaccine Wastage Rates Calculator assuming African Region, four weeks of retention of vaccine after vial opened, 2 dose schedule, 90% coverage, daily immunization sessions, and influenza vaccine.[4]
**Reserve stock**	3 months at national level<br>1 month at district and regional levels<br>0.5 months at health facility level	No reserve stock	Vaccine reserve stock are the excess supply in case of increased demand or stock-outs. Routine inputs are from WHO guidance while SARS-CoV-2 inputs assume high global demand and limited supply.[5-7]
**Resupply intervals**	3 months at national, district, and regional levels<br>1 month at health facility level	Every month	Three month supply interval is common for routine immunization in low resource settings,[8] while SARS-CoV-2 supply interval assumes high global demand and limited supply.
**Vaccinators**	Nurse density per capita for WHO African Region countries multiplied by the simulated country population, the proportion of nurses providing immunization services, and estimates of absenteeism at baseline and during the SARS-CoV-2 pandemic	Same as for routine	Typically, persons delivering vaccines in the region are nurses, but not all nurses engage in the provision of immunization services. The estimates for nurses per capita are from WHO.[9] Estimates of percentage of nurses providing immunization services are from the Organisation for Economic Co-operation and Development.[10] Absenteeism estimates from observational data (baseline) and modelling data (SARS-CoV-2) from the United States.[11,12]

Supplemental Table 3. African Region routine immunization programs as of 2017[a]

Country[b]	MCV2	PCV	Rota	HPV	Rubella	Tetanus[b] age 2	Tetanus[b] adolescent	MenA	YF	BCG
1. Algeria	Y	Y	N	N	Y	N	Y	N	N	Y
2. Angola	Y	Y	Y	N	N	N	N	N	Y	Y
3. Benin	N	Y	N	N	N	N	N	N	Y	Y
4. Botswana	Y	Y	Y	Y	Y	Y	N	N	N	Y
5. Burkina Faso	Y	Y	Y	N	Y	N	N	Y	Y	Y
6. Burundi	Y	Y	Y	N	Y	Y	N	N	N	Y
7. Cabo Verde	Y	N	N	N	Y	N	N	N	N	Y
8. Cameroon	N	Y	Y	N	Y	N	N	N	Y	Y
9. Central African Republic	N	Y	N	N	N	N	N	Y	Y	Y
10. Chad	N	N	N	N	N	N	N	Y	Y	Y
11. Comoros	N	N	N	N	N	N	N	N	N	Y
12. Congo	N	Y	Y	N	N	N	N	N	Y	Y
13. Côte d'Ivoire	N	Y	Y	N	N	N	N	N	Y	Y
14. Democratic Republic of the Congo	N	Y	N	N	N	N	N	N	Y	Y
15. Equatorial Guinea	N	N	N	N	N	N	N	N	Y	Y
16. Eritrea	Y	Y	Y	N	Y	N	N	N	N	Y
17. Eswatini	Y	Y	Y	N	Y	N	N	N	N	Y
18. Ethiopia	N	Y	Y	N	N	N	N	N	N	Y
19. Gabon	N	N	N	N	N	N	N	N	Y	Y
20. Gambia	Y	Y	Y	N	Y	Y	N	N	Y	Y
21. Ghana	Y	Y	Y	N	Y	N	N	Y	Y	Y
22. Guinea	N	N	N	Y	N	N	N	N	Y	Y
23. Guinea-Bissau	N	Y	Y	N	N	N	N	N	Y	Y
24. Kenya	Y	Y	Y	N	Y	N	N	N	N	Y
25. Lesotho	Y	Y	Y	N	Y	Y	N	N	N	Y
26. Liberia	N	Y	Y	N	N	N	N	N	Y	Y
27. Madagascar	N	Y	Y	N	N	N	N	N	N	Y
28. Malawi	Y	Y	Y	N	Y	N	N	N	N	Y
29. Mali	N	Y	Y	N	N	N	N	Y	Y	Y
30. Mauritania	N	Y	Y	N	N	N	N	N	N	Y
31. Mauritius	Y	Y	Y	Y	Y	Y	N	N	N	N
32. Mozambique	Y	Y	Y	Y	N	N	N	N	N	Y
33. Namibia	Y	Y	Y	N	Y	N	Y	N	N	Y
34. Niger	Y	Y	Y	N	N	N	N	Y	Y	Y
35. Nigeria	N	Y	N	N	N	N	N	N	Y	Y
36. Rwanda	Y	Y	Y	Y	Y	N	N	N	N	Y
37. Sao Tome and Principe	Y	Y	Y	Y	Y	N	N	N	Y	Y
38. Senegal	Y	Y	Y	N	Y	N	N	N	Y	Y
39. Seychelles	Y	N	Y	Y	Y	Y	N	N	N	Y
40. Sierra Leone	Y	Y	Y	N	N	N	N	N	Y	Y
41. South Africa	Y	Y	Y	Y	N	N	Y	N	N	Y
42. South Sudan	N	N	N	N	N	N	N	N	N	Y
43. Togo	N	Y	Y	N	N	N	N	N	Y	Y
44. Uganda	N	Y	N	Y	N	N	N	N	N	Y
45. Tanzania	Y	Y	Y	N	Y	N	N	N	N	Y
46. Zambia	Y	Y	Y	N	Y	N	N	N	N	Y
47. Zimbabwe	Y	Y	Y	Y	Y	Y	N	N	N	Y
Total	26	39	34	10	22	7	3	6	23	46
%	55.3%	83.0%	72.3%	21.3%	46.8%	14.9%	6.4%	12.7%	48.9%	97.9%

Notes:

a. Tetanus-containing vaccines given at time periods noted. Some more common routine vaccines were excluded from this table, including diphtheria-tetanus-pertussis, hepatitis B, and *Haemophilus influenzae* type b vaccines. Abbreviations: MCV2=second dose of measles-containing vaccine, PCV=pneumococcal conjugate vaccine, Rota=rotavirus vaccine, HPV=human papillomavirus vaccine, MenA= Neisseria meningitidis group A vaccine, YF= yellow fever vaccine, BCG= bacille Calmette-Guérin vaccine

b. Data are from 47 African Region countries reporting 2017 data to the WHO UNICEF Joint Reporting Form (JRF) on Immunization.[13].

Supplemental Table 4. Vaccine doses and doses per vaccinator for routine and SARS-CoV-2 vaccination programs by time period by nurse density estimate for WHO Regions

Source of nurse density input	Nurse density per 10,000 population[a]	Routine doses / vaccinator[c] (baseline)	Routine doses / vaccinator (during pandemic)	≥65 years (SARS-CoV-2)		Chronic diseases (SARS-CoV-2)		HCWs (SARS-CoV-2)		All risk groups combined (SARS-CoV-2)		Comparative workload between regions
				Doses / vaccinator	% of baseline routine for African Region	Doses / vaccinator	% of baseline routine for African Region	Doses / vaccinator	% of baseline routine for African Region	Doses / vaccinator	% of baseline routine for African Region	% WHO Regional Office divided by simulated country SARS-CoV-2 vaccine doses / vaccinator[d]
African Region Country	6.9	160.1	174.1	52.0	32.5%	176.7	110.4%	2.2	1.4%	199.9	124.8%	--
Comparison using nurse density from other WHO Regions												
Americas	25.7	43.0	46.7	14.0	8.7%	47.4	29.6%	0.6	0.4%	53.7	33.5%	26.8%
Eastern Mediterranean	18.9	58.5	63.5	19.0	11.9%	64.5	40.3%	0.8	0.5%	73.0	45.6%	36.5%
Europe	66.5	16.6	18.1	5.4	3.4%	18.3	11.5%	0.2	0.1%	20.7	13.0%	10.4%
South-East Asia	16.4	67.4	73.2	21.9	13.7%	74.4	46.4%	0.9	0.6%	84.1	52.5%	42.1%
Western Pacific	34.9	31.7	34.4	10.3	6.4%	34.9	21.8%	0.4	0.3%	39.5	24.7%	19.8%
Global	28.5	38.8	42.1	12.6	7.9%	42.8	26.7%	0.5	0.3%	48.4	30.2%	24.2%

Notes:

a. Nurse density data are from WHO Global Health Workforce Statistics. We used the median value from countries per WHO Region at the most recent available date and divided by country population estimates to calculate per capita values.[9,14]
b. Total vaccinators is the nurse density multiplied by the simulated country population (20,000,000) x 43% (the estimated percentage of nurses that provide immunization services).[9,10]
c. Adjustments account for 3% absenteeism (baseline) and an additional 8% absenteeism (during pandemic).[11,12]
d. The relationship of SARS-CoV-2 vaccine workload between the estimates for the simulated country using African Region versus other WHO Regional Office nurse density estimates is the same regardless of target group.

Supplemental Figure 1. National level monthly volumes stored for routine and SARS-CoV-2 risk group vaccination

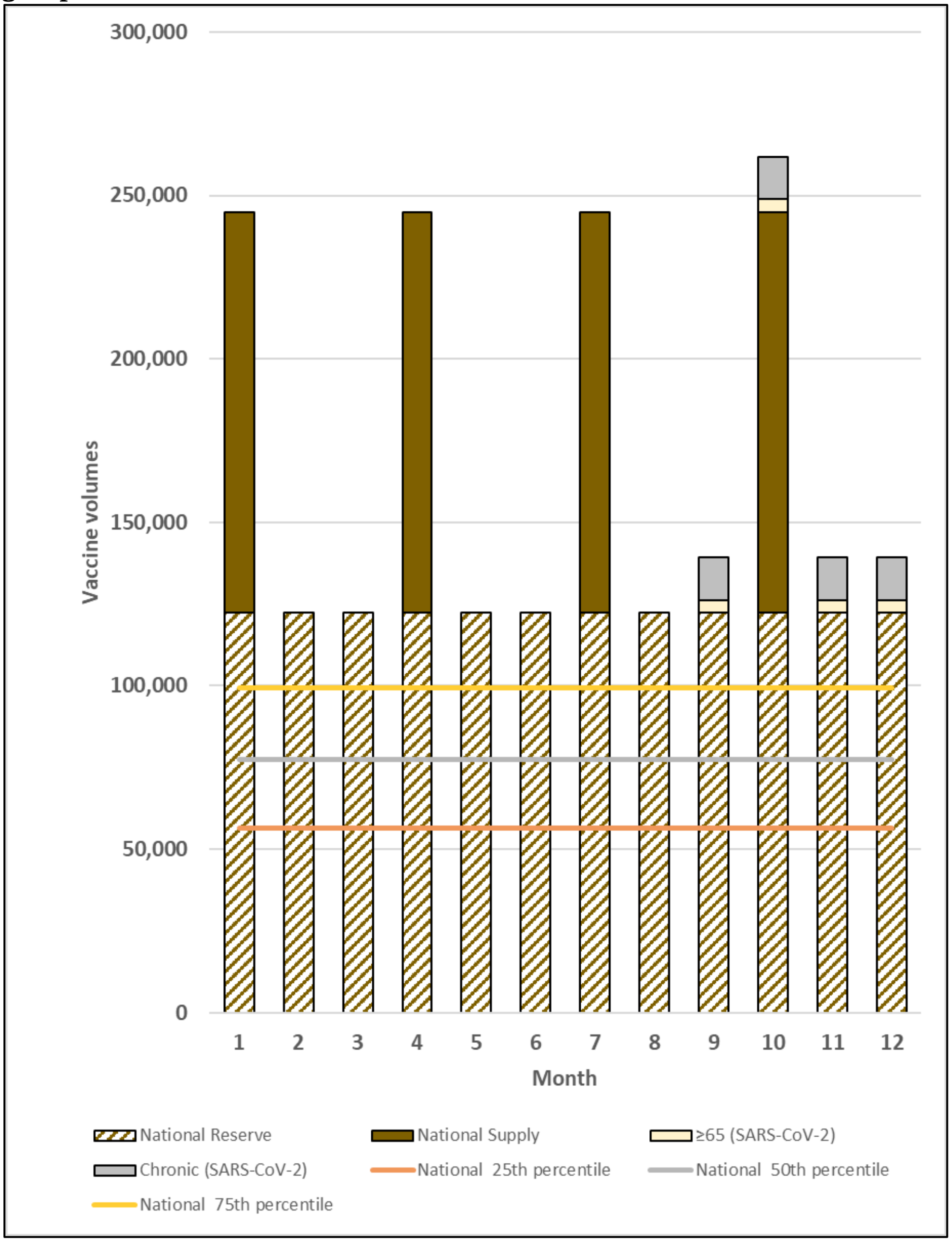

Notes:

1. Figure depicts total national level vaccine volumes calculated according to study assumptions and the vaccine flow down schematic (Figure 1).
2. The highest volume month (month 10) was chosen for the maximum national level monthly analyses.
3. HCWs target group removed because volume contribution was too small to depict in the figure.
4. Upper and lower range capacity excluded from this figure

Supplemental Figure 2. Subnational level monthly volumes stored for routine and SARS-CoV-2 risk group vaccination

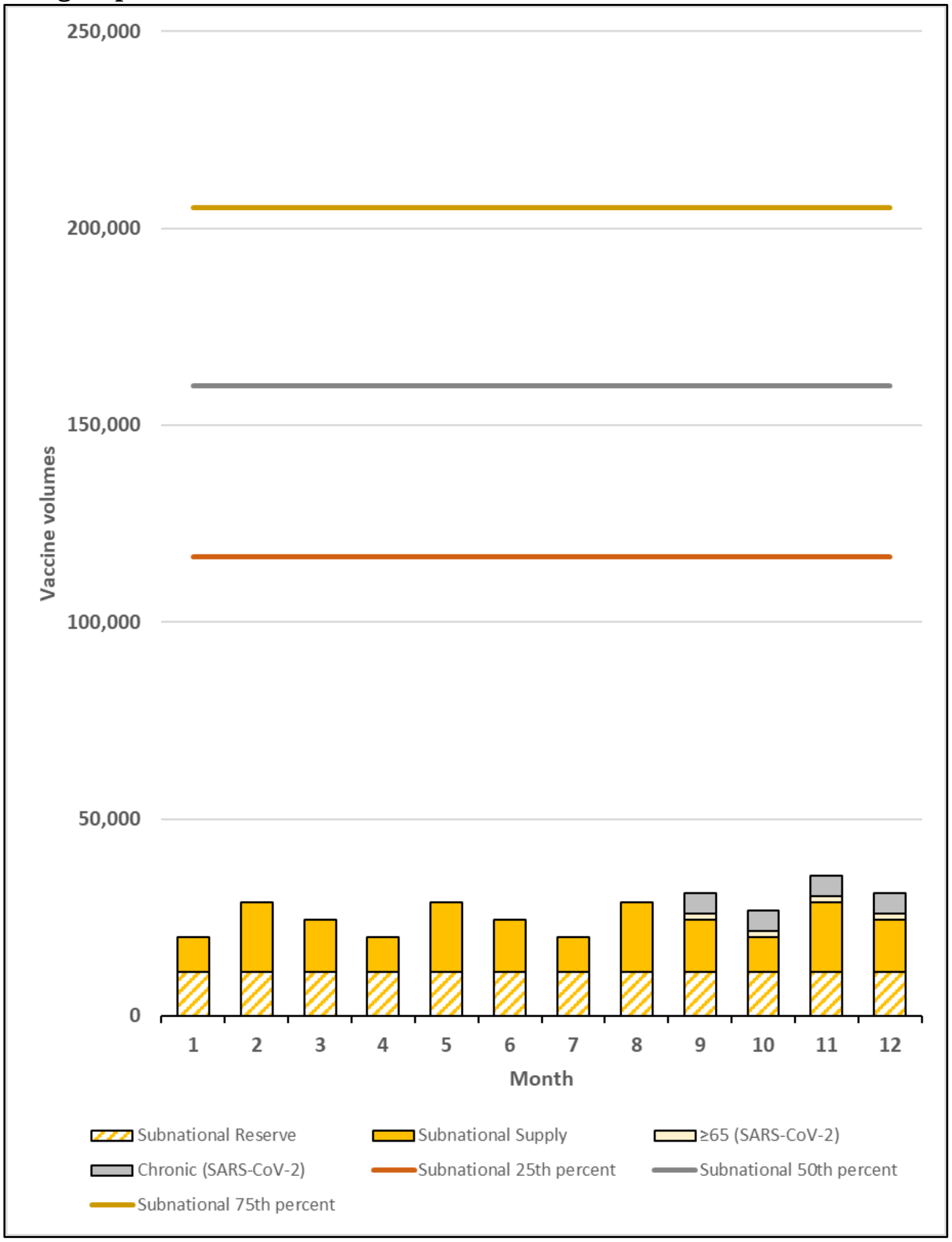

Notes:

1. Figure depicts total national level vaccine volumes calculated according to study assumptions and the vaccine flow down schematic (Figure 1).
2. The highest volume month (month 11) was chosen for the maximum national level monthly analyses.
3. HCWs target group removed because volume contribution was too small to depict in the figure.
4. Upper and lower range capacity excluded from this figure

SUPPLEMENT REFERENCES

1. World Health Organization. Table 1: Summary of WHO Position Papers - Recommendations for Routine Immunization. 2019. https://www.who.int/immunization/policy/Immunization_routine_table1.pdf (accessed December 17, 2019).
2. World Health Organization. WHO Prequalified Vaccines. 2019. https://extranet.who.int/gavi/PQ_Web/ (accessed March 22, 2019).
3. World Health Organization. A global framework to ensure equitable and fair allocation of COVID-19 products. 2020. https://apps.who.int/gb/COVID-19/pdf_files/18_06/Global%20Allocation%20Framework.pdf (accessed June 26, 2020).
4. World Health Organization. WHO Vaccine Wastage Rates Calculator. 2020. https://www.who.int/immunization/programmes_systems/supply_chain/resources/WHO_Vaccine_Wastage_Rates_Calculator_Tool.xlsx (accessed 25 April 2020).
5. World Health Organization. Vaccine Introduction Guidelines. 2005. https://www.who.int/immunization/hpv/plan/vaccine_introduction_guidelines_who_2005.pdf (accessed 02 March 2020).
6. World Health Organization. Training for mid-level managers (MLM) module 1: cold chain, vaccines and safe-injection equipment management. 2008. http://apps.who.int/iris/bitstream/10665/70184/1/WHO_IVB_08.01_eng.pdf (accessed January 4, 2020).
7. World Health Organization. Principles and considerations for adding a vaccine to a national immunization programme: from decision to implementation and monitoring. 2014. https://apps.who.int/iris/handle/10665/111548 (accessed June 29, 2020).
8. World Health Organization. Guideline for establishing or improving primary and intermediate vaccine stores. 2002. https://apps.who.int/iris/bitstream/handle/10665/67807/WHO_V-B_02.34_eng.pdf).
9. World Health Organization. WHO Global Health Workforce Statistics: December 2018 update. 2018. https://www.who.int/hrh/statistics/hwfstats/en/ (accessed January 22, 2020).
10. International Council of Nurses. The role of nurses in immunisation: a snapshot from oecd countries. 2018. https://www.icn.ch/sites/default/files/inline-files/IMMUNISATION_Report%20%28002%29.pdf (accessed June 3, 2020).
11. Chin ET, Huynh BQ, Lo NC, Hastie T, Basu S. Healthcare worker absenteeism, child care costs, and COVID-19 school closures: a simulation analysis. *medRxiv* 2020.
12. Ticharwa M, Cope V, Murray M. Nurse absenteeism: An analysis of trends and perceptions of nurse unit managers. *J Nurs Manag* 2019; **27**(1): 109-16.
13. Ortiz JR, Perut M, Dumolard L, et al. A global review of national influenza immunization policies: Analysis of the 2014 WHO/UNICEF Joint Reporting Form on immunization. *Vaccine* 2016; **34**(45): 5400-5.
14. Institute for Health Metrics and Evaluation. Global Burden of Disease Study 2017 (GBD 2017) Population Estimates 1950-2017. 2019. http://ghdx.healthdata.org/record/ihme-data/gbd-2017-population-estimates-1950-2017 (accessed December 20, 2019).
